# Influence of Electrotherapy with Task-Oriented Training on Spasticity, Hand Function, Upper Limb Function, and Activities of Daily Living in Patients with Subacute Stroke: A Double-Blinded, Randomized, Controlled Trial

**DOI:** 10.3390/healthcare9080987

**Published:** 2021-08-03

**Authors:** Jong-Hoon Moon, Hwi-Young Cho, Suk-Chan Hahm

**Affiliations:** 1 Department of Occupational Therapy, Kyungdong University, Wonju 26495, Korea; garnett231@kduniv.ac.kr; 2 Department of Physical Therapy, Gachon University, Incheon 21936, Korea; 3 Graduate School of Integrative Medicine, CHA University, Seongnam 13488, Korea

**Keywords:** activities of daily living, electrotherapy, stroke, spasticity, transcutaneous electrical nerve stimulation, upper limb function

## Abstract

The effects of electrotherapy with task-oriented training on upper limb function in subacute stroke patients are unclear. This study investigated the influence of transcutaneous electrical nerve stimulation (TENS) with task-oriented training on spasticity, hand function, upper limb function, and activities of daily living in patients with subacute stroke. Forty-eight patients with subacute stroke were randomly assigned to either the TENS group (n = 22) or the placebo-TENS group (n = 21). High-frequency (100 Hz) TENS with below-motor threshold intensity or placebo-TENS was applied for 30 min/day, five times a week, for 4 weeks. The two groups also received task-oriented training after TENS. The Modified Ashworth Scale (MAS), Jebsen–Taylor Hand Function Test (JTHFT), Manual Function Test (MFT), and Modified Barthel Index (MBI) were used to assess spasticity, hand function, upper limb function, and activities of daily living, respectively. There was a significant time–group interaction with the MFT (*p* = 0.003). The TENS group showed significantly improved MAS (*p* = 0.003), JTHFT (*p* < 0.001), MFT (*p* < 0.001), and MBI (*p* < 0.001) scores after the intervention. The placebo-TENS group showed significantly improved JTHFT (*p* < 0.001), MFT (*p* = 0.001), and MBI scores (*p* < 0.001). There was a significant correlation between the MFT and MBI scores (*p* = 0.025). These results suggest that electrotherapy with task-oriented training can be used to improve upper limb function in patients with subacute stroke.

## 1. Introduction

Stroke is a disease that involves sensory, motor, cognitive, and speech disabilities due to cerebral blood vessel disease [1]. A previous study reported that post-stroke permanent motor dysfunction is more severe in the upper limbs than in the lower limbs [2]. Disabilities in upper limb function, including hand function, lead to limitations in activities of daily living [2]. Thus, improving upper limb function and activities of daily living is an important goal of rehabilitation for patients with stroke [3].

Transcutaneous electrical nerve stimulation (TENS)—especially high-frequency TENS—is an electrical stimulation treatment used to improve motor function in patients with stroke [4]. Levin and Hui-Chan reported that TENS can reduce spasticity and improve motor function in patients with stroke [5]. These effects may be due to the plastic changes in the brain caused by the application and enhancement of TENS in the reciprocal inhibition of concentric and eccentric muscles via disinhibition of the upper motor neurons [5,6,7].

Tekeolu et al. examined the effect of TENS with therapeutic exercises for 8 weeks on the functional recovery of patients with stroke. According to their study, the TENS group showed significant improvements in activities of daily living compared with the placebo-TENS group [8]. Ikuno et al. also reported that task-oriented training and peripheral sensory nerve stimulation are effective in improving upper limb function in patients with stroke [9]. Recently, Jung et al. reported that the application of TENS during task-oriented training was effective in improving upper limb function and muscle activity in patients with chronic stroke [10]. Despite these findings, the exact effect of TENS in improving the hand function and activities of daily living in patients with stroke is still unclear. Few studies have reported the effect of combining TENS with occupational and physical therapies for the recovery of upper limb function in patients with subacute stroke. In the study of Tekeolu et al., the TENS electrode was attached to the upper and lower limbs [8]; thus, the treatment was not focused on the upper limbs. A study was also recently performed on patients with chronic stroke; however, changes in their activities of daily living were not assessed [10]. Furthermore, another study did not use electrical stimulation in the placebo-TENS group, and so did not blind the participants [9]. Thus, it is important to demonstrate the effect of TENS on the rehabilitation of patients with stroke using an appropriate placebo-TENS application method.

Therefore, the aim of this study was to investigate the influence of TENS with task-oriented training on spasticity, hand function, upper limb function, and activities of daily living in patients with subacute stroke, using an appropriate placebo-TENS application method.

## 2. Methods

### 2.1. Design and Ethics

This study was designed as a double-blinded, randomized, controlled clinical trial. This study was approved by the Institutional Review Board of Gachon University (1044396-201803-HR-073-01) and registered (WHO International Clinical Trials Registry Platform, KCT0006318). Informed consent was obtained from all subjects involved in the study.

### 2.2. Participants and Sample Size

The inclusion criteria of the participants were as follows: (1) first stroke diagnosed by a neurologist; (2) middle cerebral artery lesions; (3) stroke onset between 1 and 3 months; and (4) fair upper limb manual muscle test findings. The exclusion criteria were as follows: (1) other neurological diseases, such as degenerative diseases; (2) severe sensory deficits; (3) severe aphasia and severe neglect; and (4) severe spasticity (contracture). 

The sample size was calculated using G*Power version 3.1.9.2. (Universität Kiel, Kiel, Germany). In this study, the effect size was set to 0.25 (medium effect size) [11]. A total of 38 participants were required to achieve 80% power at an α-level of 0.05. Considering a dropout rate of 20%, a total sample size of 48 participants was determined.

### 2.3. Randomization and Blinding

Participants were randomly allocated to either the TENS or placebo-TENS groups using the block randomization method [12]. To conceal the treatment allocation, no group information was provided to either the assessor or the data analyst. Subjects, therapists, and assessors were asked not to discuss their allocations.

### 2.4. Intervention

In the TENS group, TENS was applied for 30 min before occupational therapy. Electrical stimulation (100 Hz, 200 μs) below the motor threshold was applied to the triceps muscle and wrist extensor muscle belly using a 2-channel TENS unit (TENS-7000, Koalaty Products Inc., Austin, TX, USA). Stimulation was applied to the level at which muscle contraction was observed. In the placebo-TENS group, electrodes were attached to the same locations, and a transient current was delivered for 30 s, then ramped down to zero over 15 s [13]. TENS was applied by a physical therapist not involved in this study.

All participants in this study received occupational and physical therapy. Occupational therapy with task-oriented training using stacking cones, rings, putty, ROM arcs, pegboards, coins, and towels was conducted. The task-oriented training was repeated for three categories: gross movement, grip, and pinch. The subjects were trained for 10 min per category and allowed to rest if they experienced fatigue. The training intensity of the tasks gradually increased after setting goals according to each subject’s athletic performance. Physical therapy—such as walking, stretching, and lower limb muscle-strengthening exercises—was also performed in both groups. Occupational and physical therapy were each performed for 30 min a day, 5 times a week, for 4 weeks.

### 2.5. Assessments

Participants were assigned to two groups through randomization after pre-assessment. Post-assessments were performed 1 d after the last intervention. All assessments were performed by investigators with more than 5 years of clinical experience and research experience with a master’s degree.

The Modified Ashworth Scale (MAS) was used to assess elbow joint spasticity. The MAS consists of six stages, from “normal” to “passive movement is impossible”. In this study, the coding used for statistical analysis ranged from 0 (normal) to 5 (rigidity). To minimize the assessment error, the assessor was completely familiar with the pre-evaluation method. The inter-rater reliability of MAS to quantify the severity of upper extremity spasticity in stroke patients was 0.69 [14].

The Jebsen–Taylor Hand Function Test (JTHFT) was used to assess hand function. The reliability of the JTHFT for the dominant hand was 0.67–0.99, while for the non-dominant hand it was 0.60–0.92 [15]. In this study, only the results for the affected side were recorded. The total score was used in the Korean version of the JTHFT. Scores range from 0 to 105 points—the higher the score, the greater the hand function [16].

The Manual Function Test (MFT) was used to assess upper limb function. This test consists of arm motions (four scales), gripping (two scales), and hand manipulation (two scales), which can identify the fine and gross motor functions of the upper limbs. The inter- and intra-rater reliabilities of the MFT were both 0.95 [17].

The Modified Barthel Index (MBI) was used to assess the activities of daily living. The scores from the evaluation of 10 activities were combined for a total of 0–100 points—the higher the score, the greater the activities of daily living. The inter-rater reliability of the MBI was 0.93–0.98, and the intra-rater reliability was 0.97–1.00 [18].

### 2.6. Statistical Analysis

Data analyses were performed using IBM SPSS (version 21.0; IBM SPSS, Chicago, IL, USA). The independent *t*-test or chi-squared test was conducted to analyze the general characteristics between the two groups. A mixed ANOVA was performed to investigate the time and group intervention for each variable. The paired *t*-test was used to compare the changes before and after the intervention in each group. Pearson’s correlation analysis was used to confirm the correlation between the MBI, MFT, JHFT, and MAS findings. A *p*-value of < 0.05 was considered statistically significant.

## 3. Results

### 3.1. Characteristics of Participants

**Figure 1:**
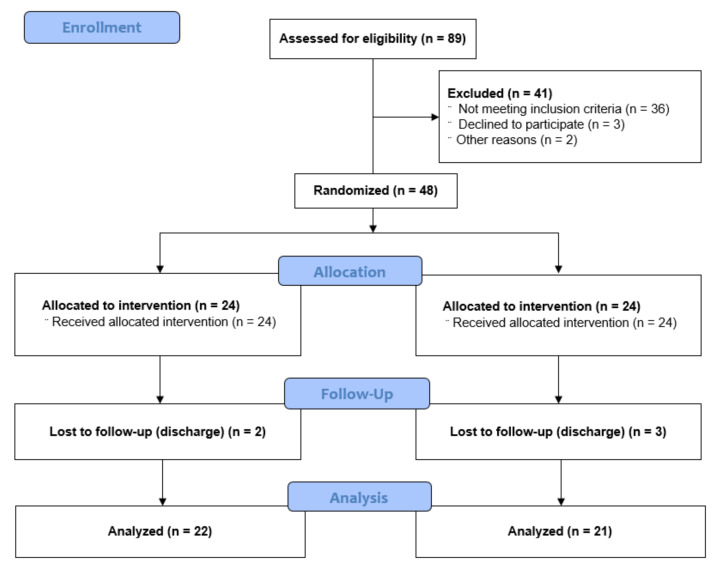
Flow diagram of the participants.

Of the 89 patients who participated in this study, 41 did not meet the inclusion criteria. The 48 subjects were randomly assigned to either the TENS group (n = 24) or the placebo-TENS group (n = 24) after pre-evaluation. Two subjects in the TENS group and three subjects in the placebo-TENS group dropped out during the study. A total of 43 subjects completed the experiment (Figure 1).

The general characteristics of the two groups are presented in Table 1. Before the intervention, there were no significant differences in the general characteristics or in the MAS, JHFT, MFT, and MBI scores between the two groups.

### 3.2. Changes in Spasticity

Comparing MAS score changes, there were no significant time–group interactions (Table 2). The TENS group showed a significant improvement in MAS scores before and after intervention (*p* = 0.003), but the placebo-TENS group did not show a significant improvement in MAS scores (Table 3). In the correlation analysis, MAS scores were not significantly associated with the scores for activities of daily living (Table 4).

### 3.3. Changes in Hand Function

Hand function, assessed using the JTHFT, did not show a significant time–group interaction (Table 2). As shown in Table 3, the TENS group showed a significant improvement in JTHFT scores before and after intervention (*p* < 0.001), as did the placebo-TENS group (*p* < 0.001). In the correlation analysis, JTHFT scores were not significantly associated with the scores for activities of daily living (Table 4).

### 3.4. Changes in Upper Limb Function

Comparing changes in MFT scores between the two groups according to time, the TENS group showed a significant time–group interaction (*p* = 0.003) (Table 2). As shown in Table 3, the TENS group showed a significant improvement in MFT scores before and after intervention (*p* < 0.001), and the placebo-TENS group also showed significant improvements in upper limb function (*p* = 0.001). In the correlation analysis, MFT scores showed a significant correlation (r = 0.341, *p* = 0.025) with the scores for activities of daily living (Table 4).

### 3.5. Changes in Activities of Daily Living

Activities of daily living, assessed using the MBI, did not show a significant time–group interaction (Table 2). As shown in Table 3, the TENS group showed a significant improvement in MBI scores before and after intervention (*p* < 0.001), as did the placebo-TENS group (*p* < 0.001).

## 4. Discussion

This study investigated the influence of TENS with task-oriented training on spasticity, hand function, upper limb function, and activities of daily living in patients with subacute stroke. There was a significant time–group interaction for upper limb function. Both the TENS and the placebo-TENS groups showed significant improvements in hand function, upper limb function, and activities of daily living after the intervention. We also found significant correlations between upper limb function and activities of daily living. Thus, TENS with task-oriented training can be used to improve upper limb function in patients with subacute stroke.

A previous systematic review reported strong evidence for the use of TENS in reducing lower limb spasticity in stroke patients [19]. However, the review did not conclude the effectiveness of TENS for upper limb spasticity because there were limited studies on TENS for upper limb spasticity in stroke patients. This study, with an appropriate placebo group to minimize the risk of bias, provides evidence of the clinical use of TENS for the management of upper limb spasticity in stroke patients. In this study, the TENS group received continuous electrical stimulation for 30 min, while the placebo-TENS group received transient current for 30 s that ramped down to zero over 15 s. Of the participants in the placebo group, 48% recognized that they were in the placebo-TENS group. Considering that the probability of guessing correctly was 50% (1:1), appropriate blinding was applied. In a previous study using the same blinding method, 57% of the participants correctly identified that they had received placebo-TENS, and the participant blinding was judged as adequate [20]. If the participant blinding had been ineffective, the percentage of subjects correctly identified as being in the placebo-TENS group would have been closer to 100%.

Spasticity was significantly decreased in the TENS group. Spasticity in the placebo-TENS group did not significantly improve after the intervention. Spasticity is caused by an increased excitability of the stretch reflexes following stroke, which is caused by the lack of function of the interneurons that transmit inhibitory effects, leading to problems in regulating reciprocal inhibition [21]. In addition, previous animal studies have reported that TENS—especially high-frequency TENS (100 Hz)—reduces spasticity following spinal cord injury and neuropathic pain following peripheral neuropathy by inhibiting glial activity in the spinal segments, which increase the excitability of the spinal neurons [22,23]. Considering that we selected a TENS frequency of 100 Hz, inhibition of the spinal glial cells by TENS may alleviate spasticity following stroke [24]. In addition, a decrease in spasticity as a result of TENS may be associated with the activation of opioid receptors in the supraspinal area. A previous study demonstrated that opioid receptors in the rostral ventral medulla decrease hyperalgesia [25]. TENS activates opioid receptors in the supraspinal area, which reduces excitability in the nervous system, and may have contributed to the reported decrease in spasticity.

Previous studies have demonstrated the effect of TENS on the functional recovery of patients with stroke. Ikuno et al. reported significantly greater improvements in upper limb function in patients with subacute stroke using task-oriented training with peripheral sensory nerve stimulation than with task-oriented training alone [9]. Jung et al. found that task-related training combined with TENS produced significantly greater improvements in upper limb function than were seen in the placebo-TENS group [10]. Our study also demonstrated the effect of TENS with task-oriented training in improving upper limb function compared with placebo-TENS with task-oriented training. A previous study did not use electrical stimulation as a placebo-TENS, and failed to blind their participants to TENS application [10]. However, we used a double-blinded design in the placebo-TENS group, and demonstrated that the TENS group showed a significant improvement in upper limb function compared with the placebo-TENS group. Thus, our data support the use of TENS for the recovery of upper limb function in patients with subacute stroke. The placebo-TENS group also showed significant improvements in upper limb function, which may be due to up to 70% of the total recovery within 1 month after the onset of stroke [26,27].

In our study, there was no difference in the activities of daily living between the two groups. However, activities of daily living showed a higher correlation with lower limb functions—such as balance [28], trunk control [29], and gait [30]—than with upper limb functions. In addition, the upper limb function-related scale items in the MBI only included grooming and feeding, and the combined score for these activities was 15 points. In other words, the proportion of upper limb function-related scores in the MBI is lower than that of lower limb function-related scores. When taken together, the MBI may not fully represent changes in the activities of daily living, as reflected by changes in upper limb function after TENS application. Despite these results, the change in MBI scores in the TENS group was 18.95 ± 11.80, while in the placebo-TENS group it was 13.86 ± 8.57. The change in the TENS group was approximately 1.5 times greater than that in the placebo-TENS group. In addition, the correlation analysis of the present study showed a significant correlation between upper limb function and activities of daily living. Thus, TENS with task-oriented training may improve activities of daily living by improving upper limb function.

A variety of interventions—including robot-assisted therapy [31], virtual reality therapy [32], mirror therapy [33], neurodevelopmental treatment [34], and botulinum toxin [35]—are being used to improve upper extremity function in stroke patients. However, robotic therapy and virtual reality therapy require expensive equipment and have limited treatment environments, while mirror therapy requires a moderate level of spatial attention, and is difficult to apply to individuals with cognitive dysfunction. Neurodevelopmental treatment may show different improvements in patient function, depending on the clinician’s proficiency with the therapeutic techniques. Botulinum toxin injection is effective for people with moderate to severe upper extremity spasticity, but it is expensive, and may have adverse effects. In contrast, TENS has no reported adverse effects, can be used as a portable device, is inexpensive, and can be applied simply, making it easy for clinical use. In addition to upper extremity training, TENS can be applied in clinics before occupational therapy, to triceps and wrist extensor muscles, in order to improve upper limb function in stroke patients.

This study had some limitations. First, only clinical assessments were performed. To quantify upper limb function, physiological responses, electromyography, or kinematic analysis may be required. Second, a follow-up assessment was not performed; thus, it is not clear how long the effect of TENS on the dependent variables lasts. Third, this study did not restrict the participants’ medications, which may have affected the study’s outcomes. Fourth, the intervention period of 4 weeks was relatively short. Finally, we did not confirm the persistent effects of TENS by conducting follow-up assessments. In the future, these limitations should be addressed, and further studies should be conducted.

## 5. Conclusions

This study demonstrated that TENS with task-oriented training improved spasticity and upper limb function in patients with subacute stroke. Activities of daily living were significantly correlated with upper limb function. Taken together, improvements in upper limb function by the application of TENS may have a positive impact on activities of daily living. Therefore, clinicians can select TENS with task-oriented training for the rehabilitation of patients with subacute stroke.

## Figures and Tables

**Table 1:** Comparison of general characteristics between the two groups.

	TENS (n = 22)	Placebo-TENS (n = 21)	*p*-Value
Sex (female/male)	13/9	10/11	0.451
Age (years) ^a^	61.23 ± 7.24	61.62 ± 8.32	0.870
Etiology (infarction/hemorrhage)	18/4	18/3	0.729
Lesion location (frontal/parietal/temporal/occipital/thalamus/basal ganglia/internal capsule/mixed lesion)	2/3/2/0/ 3/5/3/4	1/4/1/1/ 4/3/4/3	0.910
Lesion side (right/left)	15/7	15/6	0.817
MMSE ^a^	27.09 ± 2.16	27.67 ± 2.08	0.379
Onset period (days) ^a^	59.41 ± 16.77	57.95 ± 15.33	0.768

^a^ Values are expressed as number of participants or mean ± SD. Abbreviations—MMSE: mini-mental state examination; TENS: transcutaneous electrical nerve stimulation.

**Table 2:** Changes in spasticity, hand function, upper limb function, and activities of daily living for time and intervention.

	TENS (n = 22)	Placebo-TENS (n = 21)		*p*-Value
MAS	−0.55 ± 0.67	−0.24 ± 0.54	Time	<0.001 *
			Group	0.103
			T × G	0.106
JTHFT	14.82 ± 7.54	10.67 ± 8.02	Time	<0.001 *
			Group	0.174
			T × G	0.088
MFT	5.59 ± 4.22	2.29 ± 2.51	Time	<0.001 *
			Group	0.101
			T × G	0.003 *
MBI	18.96 ± 11.80	13.86 ± 8.57	Time	<0.001 *
			Group	0.301
			T × G	0.114

Values are expressed as mean ± SD for post values–pre values. Abbreviations—TENS: transcutaneous electrical nerve stimulation; MAS: Modified Ashworth Scale; JTHFT: Jebsen–Taylor Hand Function Test; MFT: Manual Function Test; MBI: Modified Barthel Index; T × G: time–group Interaction. * *p* < 0.05.

**Table 3:** Changes in spasticity, hand function, upper limb function, and activities of daily living for each group.

	TENS (n = 22)			Placebo-TENS (n = 21)		
	Pre	Post	*p*-Value	Pre	Post	*p*-Value
MAS	1.23 ± 0.53	0.68 ± 0.57	0.003 *	1.29 ± 0.46	1.05 ± 0.50	0.059
JTHFT	26.32 ± 8.48	41.14 ± 8.79	<0.001 *	24.71 ± 9.74	35.38 ± 11.03	<0.001 *
MFT	13.27 ± 3.47	18.86 ± 3.83	<0.001 *	13.38 ± 3.03	15.67 ± 3.55	0.001 *
MBI	52.50 ± 11.01	71.46 ± 12.95	<0.001 *	51.43 ± 12.30	65.29 ± 13.49	<0.001 *

Values are expressed as mean ± SD. Abbreviations—TENS: transcutaneous electrical nerve stimulation; MAS: Modified Ashworth Scale; JTHFT: Jebsen–Taylor Hand Function Test; MFT: Manual Function Test; MBI: Modified Barthel Index. * *p* < 0.05.

**Table 4:** Correlation of spasticity, hand function, or upper limb function with activities of daily living.

	MBI	
	r	*p*-Value
MAS	−0.087	0.578
JTHFT	0.167	0.284
MFT	0.341	0.025 *

Abbreviations—MAS: Modified Ashworth Scale; JHFT: Jebsen–Taylor Hand Function Test; MFT: Manual Function Test; MBI: Modified Barthel Index. * *p* < 0.05.

## Data Availability

The data presented in this study are available upon request from the corresponding author.
